# Higher mitochondrial DNA copy number is associated with metformin-induced weight loss

**DOI:** 10.1038/s43856-023-00258-0

**Published:** 2023-02-18

**Authors:** Jing Wang, Hua Liang, Rong Huang, Xiong Weng, Li Zheng, You Wang, Xueying Zheng, Zhenglong Gu, Fei Chen, Jian Shao, Zhaoxu Geng, Ewan R. Pearson, Jianping Weng, Wenying Yang, Tao Xu, Kaixin Zhou

**Affiliations:** 1 College of Life Sciences, University of Chinese Academy of Sciences, Beijing, China; 2 Department of Endocrinology and Metabolism, The Third Affiliated Hospital, Sun Yat-sen University, and Guangdong Provincial Key Laboratory of Diabetology, Guangzhou, 510630 Guangdong, China; 3 Department of Endocrinology and Metabolism, Shunde Hospital of Southern Medical University (The First People’s Hospital of Shunde), No. 1 Jiazi Road, Lunjiao Street, Foshan, 528300 P. R. China; 4 Medical Science and Technology Innovation Center, Jinan Central Hospital, Shandong First Medical University, Jinan, 250013 Shandong China; 5 Division of Systems Medicine, School of Medicine, University of Dundee, Dundee, UK; 6 Institute of Biophysics, Chinese Academy of Sciences, Beijing, China; 7 Department of Endocrinology, Institute of Endocrine and Metabolic Diseases, the First Affiliated Hospital of USTC, Division of Life Sciences and Medicine, University of Science and Technology of China, Hefei, Anhui 230001 China; 8 Center for Mitochondrial Genetics and Health, Greater Bay Area Institute of Precision Medicine, Guangzhou, China; 9 School of Life Sciences, Fudan University, Shanghai, China; 10 Population Health and Genomics, School of Medicine, University of Dundee, Dundee, UK; 11 Department of Endocrinology, China-Japan Friendship Hospital, Beijing, 100029 China; 12 Guangzhou Laboratory, Guangzhou, China

**Keywords:** Mitochondrial genome, Predictive markers

## Abstract

**Background:**

Considerable variability exists in response to metformin with few effective biomarkers to guide the treatment. Here we evaluated whether whole blood derived mitochondrial DNA copy number (mtDNA-CN) is a biomarker of metformin response as measured by glucose reduction or weight loss.

**Methods:**

Using data from the trial of Metformin (*n* = 304) and AcaRbose (*n* = 300) in Chinese as the initial Hypoglycaemic treatment (MARCH), we examined the association between mtDNA-CN and two metformin response outcomes of HbA1c reduction and weight loss. The acarbose arm was used as a comparator group. Whole blood mtDNA-CN was estimated by deep whole genome sequencing with adjustments for confounders. Multiple linear regression and repeated measurement analyses were used to evaluate the association between mtDNA-CN and drug response outcomes.

**Results:**

Here we show that glucose reduction is not significantly associated with mtDNA-CN and in either treatment arm. In the metformin arm, each increase of 1 SD in mtDNA-CN is significantly (*P* = 0.006) associated with a 0.43 kg more weight loss. Repeated measurement analysis shows that after 16 weeks of metformin monotherapy, patients in the top tertile of mtDNA-CN consistently lost 1.21 kg more weight than those in the bottom tertile (*P* < 0.001). In comparison, mtDNA-CN is not significantly associated with acarbose-induced weight loss.

**Conclusions:**

Patients with higher mtDNA-CN are likely to lose more weight upon metformin treatment, suggesting mtDNA-CN as a potential novel biomarker for more effective weight management in type 2 diabetes.

## Plain language summary

Treatment of diabetes with the drug metformin can lead to beneficial weight loss. However, there is considerable variability in how patients respond to metformin and few markers or tests are available to guide prescribing. Here, we look at data from patients who took part in a trial comparing metformin with another diabetes drug and determine whether a particular marker—mitochondrial DNA copy number (mtDNA-CN)—is associated with weight loss with these treatments. mtDNA-CN is a proxy for the function of the mitochondria, an important organelle for the generation of metabolic energy in eukaryotic cells. Our results show that patients with diabetes with a higher mtDNA-CN lost more weight upon metformin treatment. This marker could potentially be used to guide treatment with metformin. Our findings warrant further exploration of mtDNA-CN as a marker of response to other drugs.

## Abstract (web summary)

Wang, Liang, Huang et al. evaluate associations between mitochondrial DNA copy number (mtDNA-CN) and HbA1c and weight loss in patients with type 2 diabetes treated with metformin or acarbose, as part of the MARCH study. They find that patients with higher mtDNA-CN lost more weight on metformin, but did not see the same with acarbose.

## Introduction

Metformin is globally recommended as the first-line oral glycaemic agent for type 2 diabetes^1^. In addition to its efficacy in lowering blood glucose, emerging evidence has shown a modest beneficial effect on weight loss^2,3^. However, considerable interindividual variability exists in patients’ response to metformin^4^. With the biological mechanisms of metformin action not being fully elucidated, both clinical and genomic factors have been suggested to affect how an individual responds to metformin^5^.

Mechanistically metformin is thought to act by modulating mitochondrial respiratory chain Complex I and mitochondrial glycerophosphate dehydrogenase to exert its pleiotropic effects on metabolism^6–8^. Metformin has also been reported to promote mitochondrial fission to improve mitochondrial respiration, restore the mitochondrial life cycle, and alleviate hyperglycaemia in obesity by activating AMP-activated protein kinase (AMPK)^9^. However, the exact nature of the mitochondrial interaction with metformin is still poorly characterized. Little evidence is available from human studies to demonstrate the mitochondrial functional impacts on metformin response.

Mitochondria are essential organelles generating most of the cellular adenosine triphosphate (ATP). Impaired mitochondrial function plays an important role in the development of insulin resistance and type 2 diabetes^10^. Previous genome-wide association studies (GWAS) have identified variants in both nuclear genome (e.g. rs6713865) and mitochondrial genome (e.g. MT:14124) contributing to the risk of type 2 diabetes and related phenotypes via altering mitochondrial function^11^. Given that hundreds to thousands copies of the mitochondrial genome exist in most cells, mitochondrial DNA copy number (mtDNA-CN) is another mitochondrial function marker that reflects its depletion, energy reserves, and oxidative stress^12,13^. Recent studies have demonstrated that mtDNA-CN from whole blood is associated with insulin resistance, type 2 diabetes and aging-related diseases^14–17^. However, there have been no reports linking mtDNA-CN to drug response in diabetes.

Here, we hypothesize that type 2 diabetes patients with varying levels of mitochondrial function, as indicated by mtDNA-CN, would have differential response to metformin treatment. A post hoc analysis of data from the Metformin and AcaRbose in Chinese as the initial Hypoglycaemic treatment (MARCH) trial is conducted. After estimating the mtDNA-CN from deep whole genome sequencing (WGS) data, we evaluate the association between mtDNA-CN and metformin response, with the acarbose arm of the trial as a comparator group. We find that diabetes patients with higher mtDNA-CN lost more weight upon metformin monotherapy. Our findings indicate mtDNA-CN as a potential biomarker to inform metformin treatment. More broadly, the pharmacogenomic role of mtDNA-CN should be examined in a wide spectrum of drugs with known mitochondria interaction.

## Methods

### Participants

Participants were selected from the MARCH trial (Registry number: ChiCTR-TRC-08000231), an open-label 48-weeks non-inferiority randomized trial to compare acarbose with metformin as the initial therapy in Chinese patients newly diagnosed with type 2 diabetes^18^. The detailed study protocol of the trial had been described previously^18^. Briefly, 788 patients were recruited between Nov, 2008 and June, 2011 from 11 clinical centres. After a 4-week screening and run-in phase, patients were randomly assigned (1:1) to metformin or acarbose monotherapy for 24 weeks. Add-on therapy with insulin secretagogues was given to those with inadequate glycaemic control after week 24. Metformin was started at 500 mg and titrated to 1500 mg once daily over four weeks. Acarbose was started from 50 mg once a day and titrated to 100 mg three times a day over the same period. Anthropometric and biochemical parameters were measured at baseline and whole blood samples were collected for DNA extraction at the same time. Weight was measured every 4 to 8 weeks during the follow-up. HbA1c was measured at baseline, 24 weeks, and 48 weeks. The protocol was approved by the Ethics Committee of China-Japan Friendship Hospital, Beijing, China (Approval number: 2008-28) and Institute of Biophysics, Chinese Academy of Sciences (Approval number: IBPIRB2019003). Genetics analysis as part of the protocol was approved by these institutes. Written informed consent was obtained from all patients^18^.

Each study centre obtained institutional review board approval. All the individuals involved in this study provided written informed consent for the main study and subsequent genetic investigations.

### Definitions of drug response

In this study, the primary drug response outcome was evaluated by HbA1c reduction whilst the secondary outcome was weight loss. Since some patients were treated with other add-on oral hypoglycaemic agents after 24 weeks, the main end point was taken at 24-week. As weight was measured every 4 to 8 weeks during the follow-up, we also assessed the weight loss from baseline to multiple follow-up time points for the two drugs.

### mtDNA copy number

Data from deep WGS (~30X) of whole blood DNA was used to derive the raw mtDNA-CN (Supplementary Table 1), which was defined as the ratio of the average coverage depth of the mitochondrial genome to that of the autosomal genome, as described before^19^. Mosdepth v0.3.0 was used to obtain the average coverage of autosomal DNA, and MToolBox v1.2.1 was used to obtain the coverage of mtDNA by filtering nuclear integrations of mitochondrial sequences (NUMTs) and amplification artifacts^20,21^. Detailed methods can be found in the Supplementary Methods.

After adjusting for plate ID, study centre ID and DNA extraction batch, the mtDNA-CN was significantly correlated with gender, age, white blood cell count, red blood cell count, and platelets count (Supplementary Table 2), in line with previous reports^22,23^. Therefore, the raw mtDNA-CN was regressed upon all these factors. The residuals were then subjected to an inverse normal transformation to derive the standardized mtDNA-CN for downstream drug response analyses.

### Statistical analysis

Baseline characteristics were expressed as the mean (SD) or number of participants (%) or median (IQR). Differences in quantitative variables between the two treatment arms were compared using a t-test or Kruskal–Wallis test depending on the distribution normality. Frequency of categorical variables were compared using the χ^2^ test. Univariate linear regression was used to examine the association between baseline factors and the standardized mtDNA-CN among all participants.

Multiple linear regression models were used to examine the associations between mtDNA-CN and each of the drug response outcomes. The impact of mtDNA-CN on drug response was first modelled within each treatment arm. In the whole data set, whether the randomized treatment agent modified the impact of mtDNA-CN on a treatment response was tested in the multiple linear model with a mtDNA-CN by treatment interaction term. All models included age, gender and relevant baseline parameters as covariates. In addition, the participants were grouped into tertiles (low, medium, and high) according to their mtDNA-CN levels. A two-way ANNOVA was used to assess the correlation between mtDNA-CN and repeated measurements of weight loss at multiple time points. All the statistical analyses were conducted in R v4.0.3. The statistical significance level was set at *P* < 0.05.

### Reporting summary

Further information on research design is available in the Nature Portfolio Reporting Summary linked to this article.

## Results

### Baseline characteristics

A total of 604 individuals, including 304 and 300 treated with metformin and acarbose respectively, were obtained for this study after the removal of participants with incomplete clinical data or poor mtDNA-CN data quality, as shown in Supplementary Fig. 1. Baseline characteristics of the participants remained in this study were summarized in Supplementary Table 3. Similar as described in the original publication, there was no statistically significant difference between the metformin arm and acarbose arm after data quality control. Furthermore, the baseline characteristics were not associated with standardized mtDNA-CN among the trial participants.

### No significant association between mtDNA-CN and glycaemic response

As shown in Table 1, baseline HbA1c was strongly associated with glycaemic response for both drugs. The level of mtDNA-CN was not significantly associated with 24-week glycaemic response to either metformin or acarbose, indicating mtDNA-CN was not an effective biomarker to predict the primary outcome of HbA1c reduction upon metformin or acarbose treatment.

**Table 1:** Multiple linear association between mtDNA-CN and glycaemic response at week 24.

Characteristics	Metformin arm		Acarbose arm	
	Beta (95% CI)	*P* value	Beta (95% CI)	*P* value
Standardized mtDNA-CN	−0.02 (−0.1–0.06)	0.636	0.01 (−0.07–0.09)	0.847
Female	−0.08 (−0.24–0.09)	0.352	0.12 (−0.06–0.3)	0.179
Age	0 (−0.01–0.01)	0.736	0 (−0.01–0.01)	0.566
Baseline HbA1c	0.71 (0.64–0.78)	<0.001	0.72 (0.64–0.8)	<0.001

Effect sizes were expressed as Beta (95% confidence interval).

### Higher mtDNA-CN is associated with more metformin-induced weight loss

Multiple linear regression models showed that for both drugs individuals with higher baseline weight, older age and being female tended to lose more weight (Table 2). Higher mtDNA-CN was associated with more weight loss in the metformin arm (*P* = 0.006); for each increase of 1 SD in mtDNA-CN there was a greater weight reduction of 0.43 kg (95% CI: 0.13–0.73) upon metformin monotherapy. In contrast, no significant association was detected between mtDNA-CN and weight loss in the acarbose arm. A significant interaction (*P* = 0.041) between mtDNA-CN and treatment arm was observed, suggesting mtDNA-CN might play different roles in the two treatment arms. Baseline weight was not significantly associated with mtDNA-CN in either treatment arm.

**Table 2:** Multiple linear association analysis between mtDNA-CN and weight loss at week 24.

Characteristics	Metformin arm		Acarbose arm	
	Beta (95% CI)	*P* value	Beta (95% CI)	*P* value
Standardized mtDNA-CN	0.43 (0.13–0.73)	0.006	−0.13 (−0.51–0.25)	0.502
Female	0.75 (0–1.5)	0.050	1.65 (0.69–2.61)	0.001
Age	0.03 (0–0.07)	0.048	0.05 (0–0.09)	0.044
Baseline weight	0.05 (0.02–0.09)	0.005	0.11 (0.06–0.15)	<0.001

Effect sizes were expressed as Beta (95% confidence interval).

### Repeated measurement analysis

**Fig. 1:**
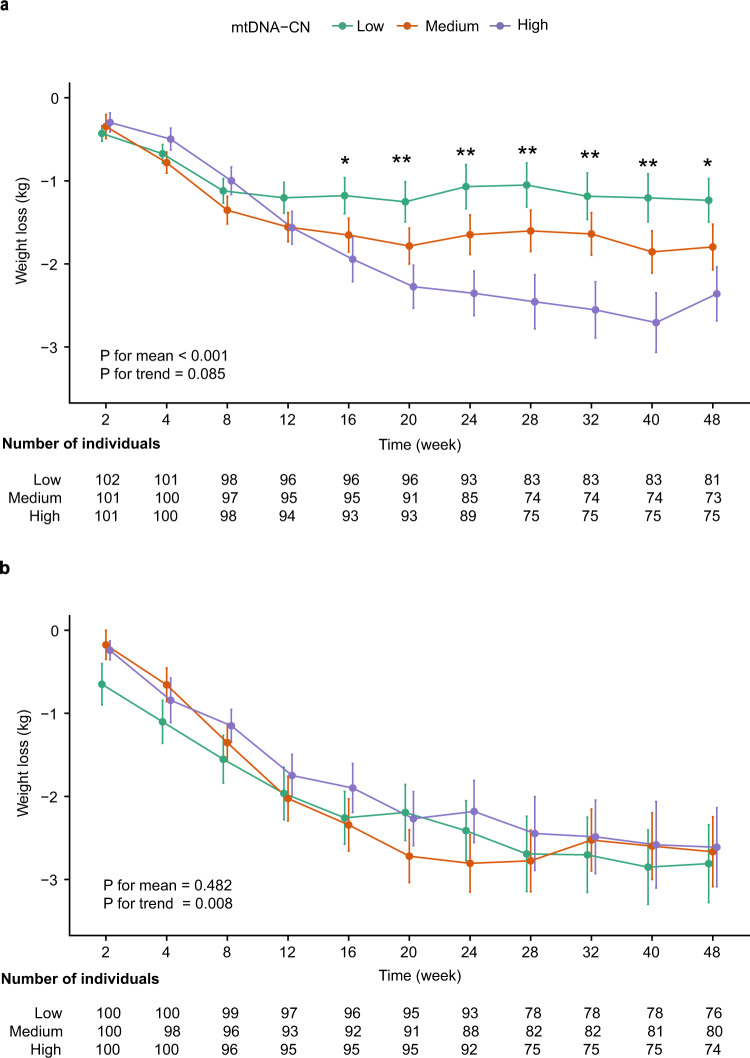
Weight loss by mitochondrial DNA copy number (mtDNA-CN) terciles at multiple time points for two drug treatment arms. **a** Metformin treatment arm. **b** Acarbose treatment arm. Error bars were standard error of the mean. Participants were evenly stratified into low (green), medium (orange), and high (purple) groups according to standardized mtDNA-CN level. *P < 0.05, **P < 0.01, *P* for three tertiles differences at 16 week (*P* = 0.038), 20 week (*P* = 0.006), 24 week (*P* = 0.001), 28 week (*P* = 0.002), 32 week (*P* = 0.005), 40 week (*P* = 0.003), 48 week (*P* = 0.018) at metformin treatment arm. *P* for mean: Repeated measurement analysis examined differences in the mean weight loss among three mtDNA-CN groups between 16 and 48 weeks of monotherapy in patients. *P* for trend: Repeated measurement analysis examined changes in weight loss over time between 16 and 48 weeks of monotherapy in patients.

To explore whether the association between mtDNA-CN and weight loss was time-dependent, comprehensive follow-up data was plotted in Fig. 1 (Supplementary Data 1). Individuals within each treatment arm were split into three tertiles according to mtDNA-CN levels. Differences in metformin-induced weight loss between the three groups only became significant after 16 weeks of treatment. Repeated measurement analysis between 16 and 48 weeks showed that higher mtDNA-CN level was significantly associated with more average weight loss (*P* < 0.001), but no significant trend of continuous weight loss during this period (*P* = 0.085). On average, patients in the top tertile lost 1.21 kg (95% CI: 0.71–1.71) more weight than those in the bottom tercile. In contrast, mtDNA-CN levels were not significantly (*P* = 0.482) associated with acarbose-induced weight loss, despite the fact that there was an overall weight benefit upon acarbose treatment. These results suggested that metformin-induced weight loss was stably associated with mtDNA-CN after 16 weeks of metformin monotherapy.

## Discussion

In this post hoc analysis of data from a randomized controlled trial, we tested the hypothesis that mitochondrial function, represented by mtDNA-CN, could be used to predict the efficacy of metformin, in terms of glycaemic response and weight loss. While no significant association between mtDNA-CN and metformin glycaemic response was observed, we found that mtDNA-CN was positively associated with metformin-induced weight loss. Individuals in the high tertile of mtDNA-CN had a consistent 1.21 kg more weight loss than those in the low tertile upon metformin monotherapy. In comparison, mtDNA-CN was associated with neither glycaemic response nor weight loss induced by acarbose.

Although the complete biological mechanisms of metformin action remain elusive, mitochondria are likely to be an important target of its action as up to a 1000-fold higher concentration of metformin could be accumulated in mitochondria compared to that in extracellular conditions^24^. It is generally recognized that mitochondria could be targeted by metformin to exert its metabolic benefit through both AMPK-dependent and AMPK-independent mechanisms^6–8^. On the other hand, metformin could also act to lower glucose without the direct involvement of mitochondria but through the Bacteroides fragilis-bile acid-intestine axis in gut^25,26^. In this study, we found positive association between mtDNA-CN and metformin-induced weight loss, strongly suggesting a key role played by mitochondrial in regulating the weight benefit of metformin. Individuals with higher mtDNA-CN probably benefited more from metformin as they had more mitochondria to interact with the drug. The contrast of mtDNA-CN impacts on metformin-induced weight loss and glucose lowering may suggest that these effects are dominated by different mechanisms, as several studies reported the effect of metformin not solely driven by glycemic control^27,28^. However, the lack of significant association between mtDNA-CN and glycaemic response does not exclude the involvement of mitochondria in glucose regulation by metformin, but may simply be due to the possibility that mitochondria are not the rate limiting step for metformin action under physiological conditions.

Without being significant absorbed, acarbose has been shown to attenuate postprandial blood glucose and induce weight loss by reversibly inhibiting α-glucosidases within the intestinal brush border, thus delaying the digestion of complex carbohydrates and disaccharides to absorbable monosaccharides^29^, or through inducing satiety and reducing food intake by enhancing the secretion of GLP-1^30,31^. Consistent with previous reports, significant glucose lowering and weight loss benefits were also observed in the acarbose arm. However, there was no significant association between mtDNA-CN and drug response in this comparator group, largely in line with the fact there is no evidence that connects mitochondria with the action of acarbose in achieving these metabolic benefits.

In this study, mtDNA-CN was captured from whole blood using the most recommended WGS platform due to its higher sensitivity and accuracy than other methods^32^. Blood-derived mtDNA-CN is associated with gene expression across multiple tissues and might indicate the metabolic health of multiple tissues in which metformin acts to promote weight loss^33^. However, the mtDNA-CN estimate might also partially reflect the haematopoiesis rather than mitochondrial function^34^. Indeed, we observed strong correlations between the raw mtDNA-CN and haematological parameters and subsequently adjusted to avoid confounding. Similarly, to avoid treatment confounding we focused on the association between baseline mtDNA-CN estimates and drug response since it has been shown metformin could improve defective haematopoiesis or increase the risk of anaemia over time^35,36^.

As the current study is a post hoc investigation of a published trial, it is largely of a hypothesis generation nature. Further replication of the findings would be required to convincingly demonstrate the potential of mtDNA-CN as a biomarker of weight response to metformin. Although this study is also limited by its modest sample size, the repeated measurements of weight spanning 48 weeks minimized the influence of random weight fluctuation. Further investigations will be informative to illustrate whether mtDNA-CN remains an effective predictor of weight loss beyond 48 weeks. Moreover, the observed association between mtDNA-CN and weight loss could also be confounded by lifestyle intervention which was not measured in this study. However, the fact that no association was observed in the comparator arm treated with acarbose makes this unlikely.

As weight management increasingly becomes an integrated goal in the treatment of type 2 diabetes^37^, the observed association between mtDNA-CN and metformin-induced weight loss is of particular interest. Lifestyle interventions and some existing glucose-lowering agents, such as metformin and SGLT2 inhibitors, have modest impacts on weight loss and are prone to weight regain. Moreover, limited biomarkers are available to explain the considerable interindividual variability in weight response, with just one pharmacogenetic study identifying multiple genetic variants weakly associated with weight loss and regain in the Diabetes Prevention Program^5^. In this study, we showed mtDNA-CN explaining 3.22% of the variance in metformin-induced weight loss, similar to that contributed by baseline weight. The clinical implication is important as the difference in weight loss of 1.21 kg between high and low mtDNA-CN tertiles is equivalent to 69% of the average weight benefit (1.75 kg) achieved by metformin therapy. Therefore, low mtDNA-CN could be an effective biomarker to identify individuals less likely to experience metformin-induced weight loss and require further intervention. For the choice of secondary weight loss options, pharmacotherapy that is mechanistically less dependent on mitochondrial function would be preferred to achieve significant and sustained weight loss.

In conclusion, higher mtDNA-CN was significantly associated with increased weight loss upon metformin monotherapy, indicating this novel type of biomarker could be valuable to inform effective weight management in type 2 diabetes patients by metformin and the choice of secondary pharmacotherapy. This study provides evidence that mtDNA-CN can be strongly associated with treatment response heterogeneity in humans. Since mitochondria are involved in the pharmacological actions of a wide spectrum of drugs^38,39^, our results raise the potential that mtDNA-CN could be a novel type of biomarker that should be examined more widely to better understand interindividual variability in drug response.

## Supplementary information

Supplementary Information

Supplementary Data 1

Description of Additional Supplementary Files

Reporting Summary

## Data Availability

Demographic and clinical details of study patients are included in Supplementary Table 3. Source data for Fig. 1 are available as Supplementary Data 1. As the part of the NyuWa genome resource, the WGS data of MARCH trial has been deposited at NODE (http://www.biosino.org/node) with accession number OEP002803. The WGS are available under restricted access due to participant consent and privacy regulations of NyuWa cohort, access can be obtained by request to the corresponding author (Kaixin Zhou: zhoukx@ucas.ac.cn), upon reasonable request.
