# Supplementary material for: Higher mitochondrial DNA copy number is associated with metformin-induced weight loss
Source: Commun Med (Lond). 2023 Feb 18;3:29. doi: 10.1038/s43856-023-00258-0 (PMC9938854; doi:10.1038/s43856-023-00258-0)
Supplement: Supplementary file 1 — Supplementary Information [file 43856_2023_258_MOESM1_ESM.pdf]

Higher mitochondrial DNA copy number is associated with metformin-induced weight loss

Jing Wang[1,12], Hua Liang[2,3,12], Rong Huang[4,12], Xiong Weng[5], Li Zheng[6], You Wang[6], Xueying Zheng[7], Zhenglong Gu[8], Fei Chen[1], Jian Shao[1], Zhaoxu Geng[6], Ewan R. Pearson[9], Jianping Weng[7], Wenying Yang[10,13], Tao Xu[6,11,13], Kaixin Zhou[1,13]

[1] College of Life Sciences, University of Chinese Academy of Sciences, Beijing, China;

[2] Department of Endocrinology and Metabolism, The Third Affiliated Hospital, Sun Yat-sen University, and Guangdong Provincial Key Laboratory of Diabetology, Guangzhou 510630, Guangdong, China;

[3] Department of Endocrinology and Metabolism, Shunde Hospital of Southern Medical University (The First People's Hospital of Shunde), No.1 Jiazi Road, Lunjiao Street, Foshan 528300, P.R.China

[4] Medical science and technology innovation center, Jinan Central Hospital, Shandong First Medical University, Jinan 250013, Shandong, China;

[5] Division of Systems Medicine, School of Medicine, University of Dundee, Dundee, UK;

[6] Institute of Biophysics, Chinese Academy of Sciences, Beijing, China;

[7] Department of Endocrinology, Institute of Endocrine and Metabolic Diseases, the First Affiliated Hospital of USTC, Division of Life Sciences and Medicine, University of Science and Technology of China, Hefei, Anhui, 230001, China;

[8] Center for Mitochondrial Genetics and Health, Greater Bay Area Institute of Precision Medicine (Guangzhou), School of Life Sciences, Fudan University, China;

[9] Population Health and Genomics, School of Medicine, University of Dundee, Dundee, U.K;

[10] Department of Endocrinology, China-Japan Friendship Hospital, Beijing 100029, China;

[11] Guangzhou Laboratory, Guangzhou, China;

[12] These authors contributed equally.

[13] These authors jointly supervised this work

Corresponding author:

Wenying Yang, ywying_1010@163.com

Tao Xu, xutao@ibp.ac.cn

Kaixin Zhou, zhoukx@ucas.ac.cn

Supplementary Figures

Supplementary Figure 1. Flow chart of quality control of the study population

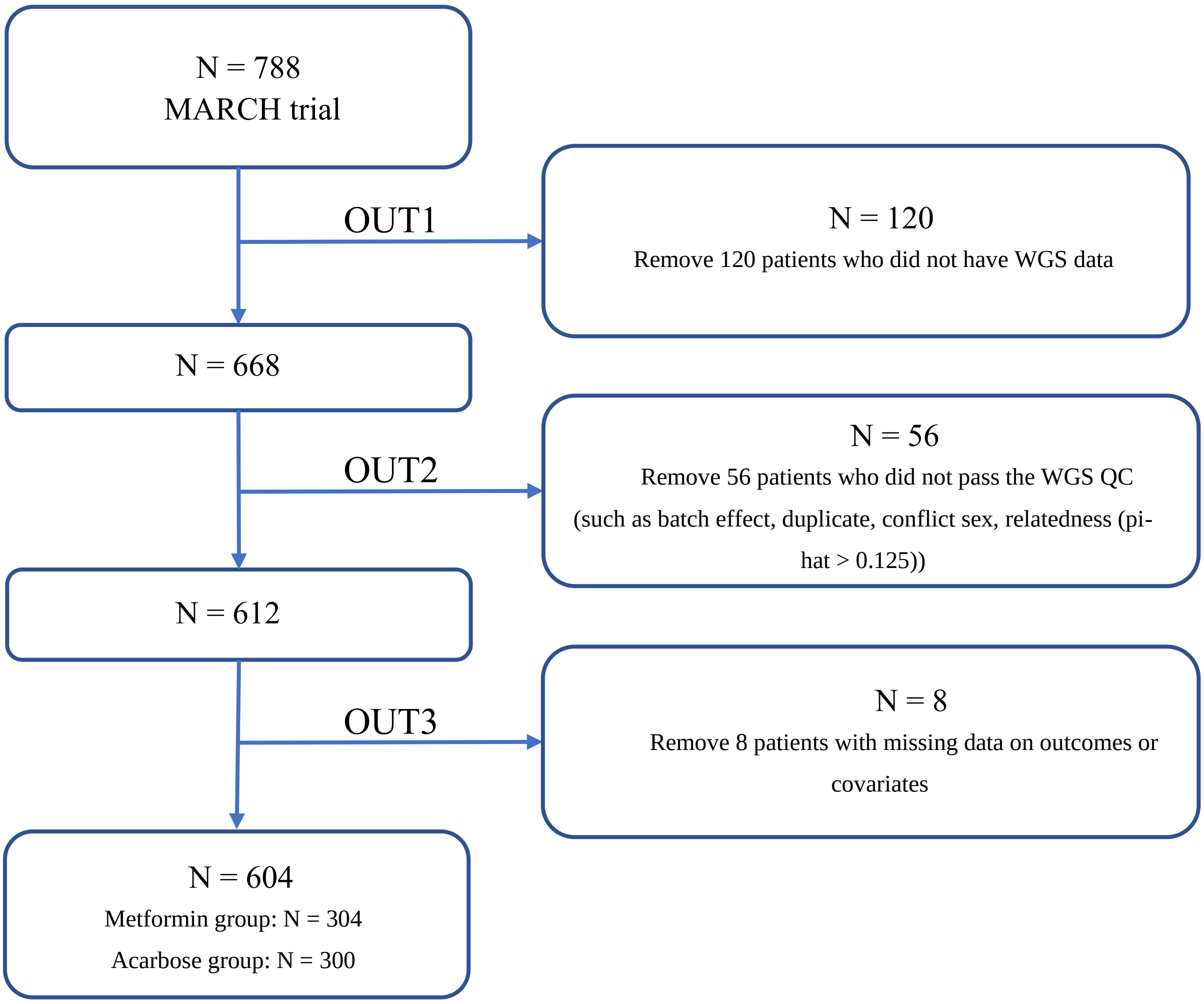

Supplementary Tables

Supplementary Table 1. The Genome characteristics of two drug treatment group

Baseline characteristics	Metformin treatment (N = 304)	Acarbose treatment (N = 300)	P
Average coverage of autosomal DNA	31.79 (5.79)	31.42 (3.45)	0.329
Average coverage of mtDNA	1785.64 (522.29)	1778.77 (539.29)	0.874
Raw estimated mtDNA-CN	112.69 (28.50)	113.35 (32.62)	0.791

Data are reported as mean (SD); P: the difference in baseline characteristics between metformin and acarbose treatment group.

Supplementary Table 2. Univariate linear regression between mtDNA-CN and gender, age, and blood cells

Baseline Characteristics	Beta (95% CI)	P value
Gender (Female)	0.09 (0.05 – 0.12)	<0.001
Age (years)	-0.82 (-1.54 – -0.1)	0.026
Platelets count (×109 cells/L)	10.9 (5.85 – 15.94)	<0.001
White blood cell count (×109 cells/L)	-0.14 (-0.27 – -0.01)	0.04
Red blood cell count (×109 cells/L)	-0.08 (-0.11 – -0.04)	<0.001

All models were adjusted for the batch effect. The effect sizes were expressed as beta (95% confidence interval).

Supplementary Table 3. Baseline characteristics of two drug treatment groups and the association between baseline characteristics and standardized mtDNA-CN

Baseline characteristics	Metformin treatment (N = 304)	Acarbose treatment (N = 300)	P*	Beta (95% CI) †	P †
Standardized mtDNA-CN	0.02 (0.96)	-0.03(1.04)	0.470	–	–
**Demographic characteristics**					
Male (%)	183 (60.20%)	185 (61.7%)	0.814	0 (-0.08–0.08)	0.923
Age (year)	50.46 (9.14)	50.33 (8.98)	0.860	0 (-0.08–0.08)	0.943
Weight (kg)	70.07 (10.69)	70.54 (10.45)	0.589	0.08 (0–0.16)	0.063
Height (cm)	165.56 (7.96)	165.65 (8.05)	0.889	0.05 (-0.03–0.13)	0.242
BMI (kg/m2)	25.63 (2.50)	25.78 (2.57)	0.470	0.07 (-0.01–0.15)	0.074
**Clinical characteristics**					
Systolic blood pressure (mm Hg)	123.04 (12.59)	122.93 (12.27)	0.909	0.02 (-0.06–0.1)	0.595
Diastolic blood pressure (mm Hg)	78.75 (7.93)	79.58 (8.45)	0.214	0.03 (-0.05–0.11)	0.464
LDL cholesterol (mmol/L)	3.00 (0.88)	3.08 (0.83)	0.238	0.02 (-0.06–0.1)	0.67
HDL cholesterol (mmol/L)	1.21 (0.27)	1.24 (0.28)	0.362	0 (-0.08–0.08)	0.941
Total cholesterol (mmol/L)	5.19 (1.01)	5.19 (0.98)	0.940	0.02 (-0.06–0.1)	0.624
Triglycerides (mmol/L)	1.92 (1.38–2.76)	1.71 (1.23–2.52)	0.055	0.05 (-0.03–0.13)	0.2
**Glycemic characteristics**					
HbA1c (%)	7.50 (1.13)	7.37 (1.08)	0.149	0.02 (-0.06–0.1)	0.573
Fasting blood glucose (mmol/L)	8.37 (1.40)	8.19 (1.38)	0.121	-0.02 (-0.1–0.06)	0.698

The data are reported as mean (SD), median (IQR) or n (%);

*: Baseline characteristics difference between metformin and acarbose arms;

†: The association between baseline characteristics and standardized mtDNA-CN in 604 participants. The effect sizes were expressed as Beta (95% confidence interval).

Supplementary methods

Whole genome sequencing and quality control

Genomic DNA was extracted from blood at baseline, and it was sequenced by Berry Genomics Co., Ltd. according to the standard protocols of NovaSeq 6000 platform. The sequencing reads were paired-end (2 × 150 nt) for each sample and the target depth is 30×. Sequencing reads quality was first checked with FastQC v0.11.3 (http://www.bioinformatics.babraham.ac.uk/projects/fastqc). Adaptor sequences and low quality bases were removed with Trimmomatic v0.36 (1). The remaining reads were mapped to human reference genome assembly 38 (Cambridge Reference Sequence for mitochondrial genome) with BWA-MEM v0.7.15 (2). Picard was used to sort the BAM files and mark duplicates. Mapping quality was check by qualimap v2.1.2 (3). Indels were realigned and bases were recalibrated according to GATK v3.7 (4). Mosdepth v0.3.0 (5) was used to obtain the average coverages for autosomal DNA. In order to exclude the influence of nuclear integrations of mitochondrial sequences (NUMTs), reads aligning to the mitochondrial genome were extracted from each BAM file and analyzed using MToolBox v1.2.1 (6) to obtain the more accurate coverages of mitochondrial genome.

Measures of mtDNA Copy Number

The raw mtDNA-CN was calculated as the ratio of the average coverage of the mitochondrial genome and the autosomal genome. This approach has been used previously (7–10). In short, this approach assumes that genomic regions of equal ploidy

should be sequenced to comparable depth. Considering that the autosomal nuclear genome is at a fixed copy number in a normal human cell, the ratio of average coverage of the mitochondrial and nuclear genomes could be used as a rough proxy of mtDNA ploidy relative to a diploid standard (10).

$$\text{mtDNA copy number} = \frac{\text{mtDNA average coverage}}{\text{Autosomal DNA average coverage}} \times 2$$

Supplementary References

1. Bolger AM, Lohse M, Usadel B. Trimmomatic: a flexible trimmer for Illumina sequence data. Bioinforma Oxf Engl. 2014 Aug 1;30(15):2114–20.

2. Li H, Durbin R. Fast and accurate long-read alignment with Burrows-Wheeler transform. Bioinforma Oxf Engl. 2010 Mar 1;26(5):589–95.

3. Okonechnikov K, Conesa A, García-Alcalde F. Qualimap 2: advanced multi-sample quality control for high-throughput sequencing data. Bioinforma Oxf Engl. 2016 Jan 15;32(2):292–4.

4. DePristo MA, Banks E, Poplin R, Garimella KV, Maguire JR, Hartl C, et al. A framework for variation discovery and genotyping using next-generation DNA sequencing data. Nat Genet. 2011;43(5):491–8.

5. Pedersen BS, Quinlan AR. Mosdepth: quick coverage calculation for genomes and exomes. Bioinformatics. 2018 Mar 1;34(5):867–8.

6. Calabrese C, Simone D, Diroma MA, Santorsola M, Guttà C, Gasparre G, et al. MToolBox: a highly automated pipeline for heteroplasmy annotation and prioritization analysis of human mitochondrial variants in high-throughput sequencing. Bioinformatics. 2014 Nov 1;30(21):3115–7.

7. Chu HT, Hsiao WW, Tsao TT, Chang CM, Liu YW, Fan CC, et al. Quantitative assessment of mitochondrial DNA copies from whole genome sequencing. BMC Genomics. 2012 Dec 13;13(7):S5.

8. Samuels DC, Li C, Li B, Song Z, Torstenson E, Boyd Clay H, et al. Recurrent Tissue-Specific mtDNA Mutations Are Common in Humans. PLoS Genet [Internet]. 2013 Nov 7 [cited 2021 Jan 11];9(11). Available from: https://www.ncbi.nlm.nih.gov/pmc/articles/PMC3820769/

9. Ding J, Sidore C, Butler TJ, Wing MK, Qian Y, Meirelles O, et al. Assessing Mitochondrial DNA Variation and Copy Number in Lymphocytes of ~2,000 Sardinians Using Tailored Sequencing Analysis Tools. PLOS Genet. 2015 Jul 14;11(7):e1005306.

10. Reznik E, Miller ML, Şenbabaoğlu Y, Riaz N, Sarungbam J, Tickoo SK, et al. Mitochondrial DNA copy number variation across human cancers. Dang CV, editor. eLife. 2016 Feb 22;5:e10769.
