# Supplementary material for: Higher mitochondrial DNA copy number is associated with metformin-induced weight loss
Source: Commun Med (Lond). 2023 Feb 18;3:29. doi: 10.1038/s43856-023-00258-0 (PMC9938854; doi:10.1038/s43856-023-00258-0)
Supplement: Supplementary file 3 — Description of Additional Supplementary Files [file 43856_2023_258_MOESM3_ESM.pdf]

Description of Additional Supplementary Files

File Name: Supplementary Data 1

Description: Source data for Figure 1
