# Supplementary material for: Higher mitochondrial DNA copy number is associated with metformin-induced weight loss
Source: Commun Med (Lond). 2023 Feb 18;3:29. doi: 10.1038/s43856-023-00258-0 (PMC9938854; doi:10.1038/s43856-023-00258-0)
Supplement: Supplementary file 4 — Reporting Summary [file 43856_2023_258_MOESM4_ESM.pdf]

nature research

Corresponding author(s): Kaixin Zhou
Last updated by author(s): Dec 21, 2022

Reporting Summary

Nature Research wishes to improve the reproducibility of the work that we publish. This form provides structure for consistency and transparency in reporting. For further information on Nature Research policies, see our Editorial Policies and the Editorial Policy Checklist.

Statistics

For all statistical analyses, confirm that the following items are present in the figure legend, table legend, main text, or Methods section.

n/a	Confirmed	
☐	☒	The exact sample size (*n*) for each experimental group/condition, given as a discrete number and unit of measurement
☐	☒	A statement on whether measurements were taken from distinct samples or whether the same sample was measured repeatedly
☐	☒	The statistical test(s) used AND whether they are one- or two-sided<br>*Only common tests should be described solely by name; describe more complex techniques in the Methods section.*
☐	☒	A description of all covariates tested
☐	☒	A description of any assumptions or corrections, such as tests of normality and adjustment for multiple comparisons
☐	☒	A full description of the statistical parameters including central tendency (e.g. means) or other basic estimates (e.g. regression coefficient) AND variation (e.g. standard deviation) or associated estimates of uncertainty (e.g. confidence intervals)
☐	☒	For null hypothesis testing, the test statistic (e.g. *F*, *t*, *r*) with confidence intervals, effect sizes, degrees of freedom and *P* value noted<br>*Give P values as exact values whenever suitable.*
☒	☐	For Bayesian analysis, information on the choice of priors and Markov chain Monte Carlo settings
☒	☐	For hierarchical and complex designs, identification of the appropriate level for tests and full reporting of outcomes
☒	☐	Estimates of effect sizes (e.g. Cohen's *d*, Pearson's *r*), indicating how they were calculated

Our web collection on statistics for biologists contains articles on many of the points above.

Software and code

Policy information about availability of computer code

Data collection	SAS version 9.10
Data analysis	R v4.0.3.

For manuscripts utilizing custom algorithms or software that are central to the research but not yet described in published literature, software must be made available to editors and reviewers. We strongly encourage code deposition in a community repository (e.g. GitHub). See the Nature Research guidelines for submitting code & software for further information.

Data

Policy information about availability of data

All manuscripts must include a data availability statement. This statement should provide the following information, where applicable:
- Accession codes, unique identifiers, or web links for publicly available datasets
- A list of figures that have associated raw data
- A description of any restrictions on data availability

Demographic and clinical details of study patients are included in table 1. Further data that support the findings of this study are available from the corresponding authors upon reasonable request following ethical approval.

Field-specific reporting

Please select the one below that is the best fit for your research. If you are not sure, read the appropriate sections before making your selection.

☒ Life sciences ☐ Behavioural & social sciences ☐ Ecological, evolutionary & environmental sciences

For a reference copy of the document with all sections, see nature.com/documents/nr-reporting-summary-flat.pdf

Life sciences study design

All studies must disclose on these points even when the disclosure is negative.

Sample size	788
Data exclusions	1) Remove 120 patients who did not have whole genotype sequencing data;2) Remove 79 patients who did not pass the genotype quality control; 3) Remove 8 patients with missing data on outcomes or covariates.
Replication	Although we did not replicate with another cohort, we analyzed repeated measurements from multiple time points in the march trial and found their results to be consistent.
Randomization	Randomisation codes were generated with a computer programme (SAS version 9.10) for eligible patients with FPG between 7·0 and 11·1 mmol/L. Patients were randomly assigned (1:1) to each of the two treatment groups (block size 8) at 11 centres.
Blinding	Neither patients nor investigators involved in the study were masked to treatment allocation. As the patients were recruited from 11 clinical sites in China, and it is difficult to achieve double-blind in many clinics. In addition, as a post hoc analysis, we focused on the association between metformin (one of the drug treatment groups of the trial) and mitochondrial DNA copy number. Therefore, the trial had little impact on the results of this study by whether it was double-blinded or not.

Reporting for specific materials, systems and methods

We require information from authors about some types of materials, experimental systems and methods used in many studies. Here, indicate whether each material, system or method listed is relevant to your study. If you are not sure if a list item applies to your research, read the appropriate section before selecting a response.

Materials & experimental systems

n/a	Involved in the study	
☒	☐	Antibodies
☒	☐	Eukaryotic cell lines
☒	☐	Palaeontology and archaeology
☒	☐	Animals and other organisms
☐	☒	Human research participants
☐	☒	Clinical data
☒	☐	Dual use research of concern

Methods

n/a	Involved in the study	
☒	☐	ChIP-seq
☒	☐	Flow cytometry
☒	☐	MRI-based neuroimaging

Human research participants

Policy information about studies involving human research participants

Population characteristics	All participants were newly diagnosed with type 2 diabetes, with a mean HbA1c of 7·5%, were enrolled from 11 sites in China. They aged from 30 to 70, both female and male. After a 4-week lifestyle modification run-in, patients were assigned to 24 weeks of monotherapy with metformin or acarbose as the initial treatment, followed by a 24-week therapy phase during which add-on therapy was used if prespecified glucose targets were not achieved.
Recruitment	Exclusion criteria :<br>1. Patients are pregnant, or are expecting to conceive within the projected duration of the study, or are breast feeding; 2. Patients unwilling to cooperate; 3. Patients with abnormal hepatic function, kidney impaired, severe chronic gastrointestinal disease, severe heart disease and evident hematological diseases; 4. Patients with clinical signs of endocrine system diseases, as thyroid diseases and hypercortisolism; 5. Patients allergic to investigational drugs; 6. Patients with mental illness or substance abuse; 7. Patients with diabetes ketoacidosis or nonketotic hyperosmolar coma requiring insulin therapy.
Ethics oversight	Medical Ethics Committee of China-Japan Friendship Hospital

Note that full information on the approval of the study protocol must also be provided in the manuscript.

Clinical data

Policy information about clinical studies

All manuscripts should comply with the ICMJE guidelines for publication of clinical research and a completed CONSORT checklist must be included with all submissions.

Clinical trial registration	This study was registered with Chinese Clinical Trial Registry, number ChiCTR-TRC-08000231
Study protocol	The full trial protocol can be accessed at the published article: DOI: 10.1016/S2213-8587(13)70021-4 (PMID: 24622668).
Data collection	Anthropometric and biochemical parameters were measured at baseline and whole blood samples were collected for DNA extraction at the same time. Weight was measured every 4 to 8 weeks during the follow-up. HbA1c was measured at baseline, 24 weeks, and 48 weeks.
Outcomes	In this study, the primary drug response outcome was evaluated by HbA1c reduction whilst the secondary outcome was weight loss. Since some patients were treated with other add-on oral hypoglycemic agents after 24 weeks, the main end point was taken at 24-week. As weight was measured every 4 to 8 weeks during the follow-up, we also assessed the weight loss from baseline to multiple follow-up time points for the two drugs.
